# What is a pilot or feasibility study? A review of current practice and editorial policy

**DOI:** 10.1186/1471-2288-10-67

**Published:** 2010-07-16

**Authors:** Mubashir Arain, Michael J Campbell, Cindy L Cooper, Gillian A Lancaster

**Affiliations:** 1 Health Services Research, ScHARR, University of Sheffield, Regent Court, Regent St Sheffield S1 4DA, UK; 2 Department of Mathematics and Statistics, University of Lancaster LA1 4YF, UK

## Abstract

**Background:**

In 2004, a review of pilot studies published in seven major medical journals during 2000-01 recommended that the statistical analysis of such studies should be either mainly descriptive or focus on sample size estimation, while results from hypothesis testing must be interpreted with caution. We revisited these journals to see whether the subsequent recommendations have changed the practice of reporting pilot studies. We also conducted a survey to identify the methodological components in registered research studies which are described as 'pilot' or 'feasibility' studies. We extended this survey to grant-awarding bodies and editors of medical journals to discover their policies regarding the function and reporting of pilot studies.

**Methods:**

Papers from 2007-08 in seven medical journals were screened to retrieve published pilot studies. Reports of registered and completed studies on the UK Clinical Research Network (UKCRN) Portfolio database were retrieved and scrutinized. Guidance on the conduct and reporting of pilot studies was retrieved from the websites of three grant giving bodies and seven journal editors were canvassed.

**Results:**

54 pilot or feasibility studies published in 2007-8 were found, of which 26 (48%) were pilot studies of interventions and the remainder feasibility studies. The majority incorporated hypothesis-testing (81%), a control arm (69%) and a randomization procedure (62%). Most (81%) pointed towards the need for further research. Only 8 out of 90 pilot studies identified by the earlier review led to subsequent main studies. Twelve studies which were interventional pilot/feasibility studies and which included testing of some component of the research process were identified through the UKCRN Portfolio database. There was no clear distinction in use of the terms 'pilot' and 'feasibility'. Five journal editors replied to our entreaty. In general they were loathe to publish studies described as 'pilot'.

**Conclusion:**

Pilot studies are still poorly reported, with inappropriate emphasis on hypothesis-testing. Authors should be aware of the different requirements of pilot studies, feasibility studies and main studies and report them appropriately. Authors should be explicit as to the purpose of a pilot study. The definitions of feasibility and pilot studies vary and we make proposals here to clarify terminology.

## Background

A brief definition is that a pilot study is a 'small study for helping to design a further confirmatory study'[1]. A very useful discussion of exactly what is a pilot study has been given by Thabane et al. [2] Such kinds of study may have various purposes such as testing study procedures, validity of tools, estimation of the recruitment rate, and estimation of parameters such as the variance of the outcome variable to calculate sample size etc. In pharmacological trials they may be referred to as 'proof of concept' or Phase I or Phase II studies. It has become apparent to us when reviewing research proposals that small studies with all the trappings of a major study, such as randomization and hypothesis testing may be labeled a 'pilot' because they do not have the power to test clinically meaningful hypotheses. The authors of such studies perhaps hope that reviewers will regard a 'pilot' more favourably than a small clinical trial. This lead us to ask when it is legitimate to label a study as a 'pilot' or 'feasibility' study, and what features should be included in these types of studies.

Lancaster et al [3] conducted a review of seven major medical journals in 2000-1 to produce evidence regarding the components of pilot studies for randomized controlled trials. Their search included both 'pilot' and 'feasibility' studies as keywords. They reported certain recommendations: having clear objectives in a pilot study, inappropriateness of mixing pilot data with main research study, using mainly descriptive statistics obtained and caution regarding the use of hypothesis testing for conclusions. Arnold et al [1] recently reviewed pilot studies particularly related to critical care medicine by searching the literature from 1997 to 2007. They provided narrative descriptions of some pilot papers particularly those describing critical care medicine procedures. They pointed out that few pilot trials later evolved into subsequent published major trials. They made useful distinctions between: *pilot work *which is any background research to inform a future study, a *pilot study *which has specific hypotheses, objectives and methodology and a *pilot trial *which is a stand-alone pilot study and includes a randomization procedure. They excluded feasibility studies from their consideration.

Thabane et al [2] gave a checklist of what they think should be included in a pilot study. They included 'feasibility' or 'vanguard' studies but did not distinguish them from pilot studies. They provided a good discussion on how to interpret a pilot study. They stress that not only the outcome or surrogate outcome for the subsequent main study should be described but also that a pilot study should have feasibility outcomes which should be clearly defined and described. Their article was opinion based and not supported by a review of current practice.

The objective of this paper is to provide writers and reviewers of research proposals with evidence from a variety of sources for which components they should expect, and which are unnecessary or unhelpful, in a study which is labeled as a pilot or feasibility study. To do this we repeated Lancaster et al's [3] review for current papers see if there has been any change in how pilot studies were reported since their study. As many pilot studies are never published we also identified pilot studies which were registered with the UK Clinical Research Network (UKCRN) Portfolio Database. This aims to be a "complete picture of the clinical research which is currently taking place across the UK". All studies included have to have been peer reviewed through a formal independent process. We examined the websites of some grant giving bodies to find their definition of a pilot study and their funding policy toward them. Finally we contacted editors of leading medical journals to discover their policy of accepting studies described as 'pilot' or 'feasibility'.

## Methods

### Literature survey

MEDLINE, Web of Science and university library data bases were searched for the years 2007-8 using the same key words "Pilot" or "Feasibility" as used by Lancaster et al. [3]. We reviewed the same four general medicine journals: the British Medical Journal (BMJ), Lancet, the New England Journal of Medicine (NEJM) and the Journal of American Medical Association (JAMA) and the same three specialist journals: British Journal of Surgery (BJS), British Journal of Cancer (BJC), British Journal of Obstetrics and Gynecology (BJOG). We excluded review papers. The full text of the relevant papers was obtained. GL reviewed 20 papers and classified them into groups as described in her original paper [3]. Subsequently MA, in discussion with MC, designed a data extraction form to classify the papers. We changed one category from GL's original paper. We separated the category 'Phase I/II trials' from the 'Piloting new treatment, technique, combination of treatments' category. We then classified the remaining paper into the categories described in Table 1. The total number of research papers by journal was obtained by searching journal article with abstracts (excluding reviews) using Pubmed. We searched citations to see whether the pilot studies identified by Lancaster et al [3] eventually led to main trials.

**Table 1:** Literature search using key words "Pilot" OR "Feasibility"

	Journal Name							
	**BMJ**	**Lancet**	**NEJM**	**JAMA**	**BJS**	**BJC**	**BJOG**	**Total**

**2007-8**								

Original articles	6	5	5	1	10	16	10	54^1 ^(1.6%)


Pilot or feasibility study in preparation for a trial	0	3	3	0	2	1	3	12

Piloting new technique, combination of treatments	1	0	0	0	4	2	6	13

Phase I, II trials	0	1	1	0	0	7	0	9

Piloting screening program	2	0	0	1	1	1	0	5

Piloting guidelines, educational package, patient care strategy	3	1	0	0	2	3	1	10

Laboratory testing of activity of compounds	0	0	1	0	1	2	0	4

Total research papers	292	379	444	383	338	1084	398	3318

**2000-1**^**2**^								

Original articles	11	17	3	7	9	33	10	90 (2.0%)

*Total research papers*	372	1115	434	619	396	1132	381	4449

^1 ^excluded Review = 8, Commentaries = 4, News = 3, Indirectly referring to previous pilot = 9

^2 ^from Lancaster et al [1]

### Portfolio database review

The (UKCRN) Portfolio Database was searched for the terms 'feasibility' or 'pilot' in the title or research summary. Duplicate cases and studies classified as 'observational' were omitted. From the remaining studies those classified as 'closed' were selected to exclude studies which may not have started or progressed. Data were extracted directly from the research summary of the database or where that was insufficient the principle investigator was contacted for related publications or study protocols.

### Editor and funding agency survey

We wrote to the seven medical journal editors of the same journals used by Lancaster et al. [3], (BMJ, Lancet, NEJM, JAMA. BJS, BJC and BJOG) and looked at the policies of three funding agencies (British Medical Research Council, Research for Patient Benefit and NETSCC (National Institute for Health Research Trials and Studies Coordinating Centre). We wished to explore whether there was any specified policy of the journal for publishing pilot trials and how the editors defined a pilot study. We also wished to see if there was funding for pilot studies.

## Results

### Literature survey

Initially 77 papers were found in the target journals for 2007-8 but 23 were review papers or commentaries or indirectly referred to the word "pilot" or "feasibility" and were not actually pilot studies leaving a total of 54 papers. Table 1 shows the results by journal and by type of study and also shows the numbers reported by Lancaster et al. [3] for 2000-01 in the same medical journals. There was a decrease in the proportion of pilot studies published over the period of time, however the difference was not statistically significant (2.0% vs 1.6%; X^2 ^= 1.6, P = 0.2). It is noticeable that the Phase I or Phase II studies are largely confined to the cancer journals.

Lancaster et al [3] found that 50% of pilot studies reported the intention of further work yet we identified only 8 (8.8%) which were followed up by a major study. Of these 2 (25%) were published in the same journal as the pilot.

Twenty-six of the studies found in 2007-8 were described as pilot or feasibility studies for randomized clinical trials (RCTs) including Phase II studies. Table 2 gives the numbers of studies which describe specific components of RCTs. Sample size calculations were performed and reported in 9 (36%) of the studies. Hypothesis testing and performing inferential statistics to report significant results was observed in 21 (81%) of pilot studies. The processes of blinding was observed in only 5 (20%) although the randomization procedure was applied or tested in 16 (62%) studies. Similarly a control group was assigned in most of the studies (n = 18; 69%). As many as 21 (81%) of pilot studies suggested the need for further investigation of the tested drug or procedure and did not report conclusive results on the basis of their pilot data. The median number of participants was 76, inter-quartile range (42, 216).

**Table 2:** Literature survey: Frequency of methodological components appearing in pilot or feasibility studies of interventions (n = 26^1^) in 2007-8

	n (%)
**Sample size calculation**	9 (35%)
**Hypothesis testing**	21 (81%)
**Randomization**	16 (62%)
**Blinding**	5 (19%)
**Control group**	18 (69%)
**Further study suggested**	21 (81%)
**Median number of participants (IQR)**	76 [8, 1299]

^1^Pilot studies = 14 Feasibility studies = 12

Of the 54 studies in 2007-8, a total of 20 were described as 'pilot' and 34 were described as 'feasibility' studies. Table 3 contrasts those which were identified by the keyword 'pilot' with those identified by 'feasibility'. Those using 'pilot' were more likely to have a pre-study sample size estimate, to use randomization and to use a control group. In the 'pilot' group 16(80%) suggested further study, in contrast to 15 (44%) in the 'feasibility' group.

**Table 3:** Literature survey: Comparison of studies (n = 54) using the key words feasibility or pilot

Study components	Pilot n = 20	Feasibility n = 34	**Chi-squared**^**1**^	P-value
Sample size	7 (35%)	3 (8%)	5.7	0.028
Hypothesis testing	14 (70%)	25 (74%)	0.78	0.51
Randomization	11 (55%)	8 (24%)	5.5	0.037
Blinding	3 (15%)	3 (9%)	0.48	0.39
Control group	13 (65%)	11 (32%)	5.4	0.020
Further study suggested	16 (80%)	15 (44%)	6.6	0.012

Number of participants Median (IQR) [Range]	62.5 (31, 189) [8, 187777]	125.5 (36, 1005) [5, 12774614]	-1.04*	0.29

^1 ^1 degree of freedom

* z-statistic (Mann-Whitney test)

### Portfolio database review

A total of 34 studies were identified using the term 'feasibility' or 'pilot' in the title or research summary which were prospective interventional studies and were closed, i.e. not currently running and available for analysis. Only 12 studies were interventional pilot/feasibility studies which included testing of some component of the research process. Of these 5 were referred to as 'feasibility', 6 as 'pilot' and 1 as both 'feasibility' and 'pilot' (Table 4).

**Table 4:** Portfolio database survey: comparison of components in studies termed pilot or feasibility

	Pilot n = 6	Feasibility n = 5	Both n = 1
*Methods related*			
Miniature RCT	4	3	0
Testing recruitment	4	1	0
Determining sample size/numbers available	3	0	1
Resources	1	0	0
Randomization	4	1	0
Outcome measures	2	4	2
Data collection	1	0	0
Follow up/dropout	2	0	0
			
*Intervention related*			
Clinical outcomes	3	1	1
Dose/efficacy/safety	0	1	0
Acceptability	2	0	1
Feasibility	3	0	1

The methodological components tested within these studies were: estimation of sample size; number of subjects eligible; resources (e.g. cost), time scale; population-related (e.g. exclusion criteria), randomisation process/acceptability; data collection systems/forms; outcome measures; follow-up (response rates, adherence); overall design; whole trial feasibility. In addition to one or more of these, some studies also looked at clinical outcomes including: feasibility/acceptability of intervention; dose, efficacy and safety of intervention.

The results are shown in Table 4. Pilot studies alone included estimation of sample size for a future bigger study and tested a greater number of components in each study. The majority of the pilots and the feasibility studies ran the whole study 'in miniature' as it would be in the full study, with or without randomization.

As an example of a pilot study consider *'CHOICES: A pilot patient preference randomised controlled trial of admission to a Women's Crisis House compared with psychiatric hospital admissions' *http://www.iop.kcl.ac.uk/projects/default.aspx?id=10290. This study looked at multiple components of a potential bigger study. It aimed to determine the proportion of women unwilling to be randomised, the feasibility of a patient preference RCT design, the outcome and cost measures to determine which outcome measures to use, the recruitment and drop out rates; and to estimate the levels of outcome variability to calculate sample sizes for the main study. It also intended to develop a user focused and designed instrument which is the outcome from the study. The sample size was 70.

### Editor and funding agency survey

The editors of five (out of seven) medical journals responded to our request for information regarding publishing policy for pilot studies. Four of the journals did not have a specified policy about publishing pilot studies and mostly reported that pilot trials cannot be published if the standard is lower than a full clinical trial requirement. The *Lancet *has started creating space for preliminary phase I trials and set a different standard for preliminary studies. Most of the other journals do not encourage the publication of pilot studies because they consider them less rigorous than main studies. Nevertheless some editors accepted pilot studies for publication by compromising only on the requirement for a pre-study sample size calculation. All other methodological issued were considered as important as for the full trials, such as trial registration, randomization, hypothesis testing, statistical analysis and reporting according to the CONSORT guidelines.

All three funding bodies made a point to note that pilot and feasibility studies would be considered for funding. Thabane et al [2] provided a list of websites which define pilot or feasibility studies. We considered the NETSCC definition to be most helpful and to most closely mirror what investigators are doing and it is given below.

**NETSCC definition of pilot and feasibility studies **http://www.netscc.ac.uk/glossary/

### Feasibility Studies

Feasibility Studies are pieces of research done before a main study. They are used to estimate important parameters that are needed to design the main study. For instance:

• standard deviation of the outcome measure, which is needed in some cases to estimate sample size,

• willingness of participants to be randomised,

• willingness of clinicians to recruit participants,

• number of eligible patients,

• characteristics of the proposed outcome measure and in some cases feasibility studies might involve designing a suitable outcome measure,

• follow-up rates, response rates to questionnaires, adherence/compliance rates, ICCs in cluster trials, etc.

Feasibility studies for randomised controlled trials may not themselves be randomised. Crucially, feasibility studies do not evaluate the outcome of interest; that is left to the main study.

If a feasibility study is a small randomised controlled trial, it need not have a primary outcome and the usual sort of power calculation is not normally undertaken. Instead the sample size should be adequate to estimate the critical parameters (e.g. recruitment rate) to the necessary degree of precision.

### Pilot studies

A Pilot Study is a version of the main study that is run in miniature to test whether the components of the main study can all work together. It is focused on the processes of the main study, for example to ensure recruitment, randomisation, treatment, and follow-up assessments all run smoothly. It will therefore resemble the main study in many respects. In some cases this will be the first phase of the substantive study and data from the pilot phase may contribute to the final analysis; this can be referred to as an internal pilot. Alternatively at the end of the pilot study the data may be analysed and set aside, a so-called external pilot.

## Discussion

In our repeat of Lancaster et al's study [3] we found that the reporting of pilot studies was still poor. It is generally accepted that small, underpowered clinical trials are unethical [4]. Thus it is not an excuse to label such a study as a pilot and hope to make it ethical. We have shown that pilot studies have different objectives to RCTs and these should be clearly described. Participants in such studies should be informed that they are in a pilot study and that there may not be a further larger study.

It is helpful to make a more formal distinction between a 'pilot' and a 'feasibility' study. We found that studies labeled 'feasibility' were conducted with more flexible methodology compared to those labeled 'pilot'. For example the term 'feasibility' has been used for large scale studies such as a screening programme applied at a population level to determine the initial feasibility of the programme. On the other hand 'pilot' studies were reported with more rigorous methodological components like sample size estimation, randomization and control group selection than studies labeled 'feasibility'. We found the NETSCC definition to be the most helpful since it distinguishes between these types of study.

In addition it was observed that most of the pilot studies report their results as inconclusive, with the intention of conducting a further, larger study. In contrast, several of the feasibility studies did not admit such an intention. On the basis of their intention one would have expected about 45 of the studies identified by Lancaster et al in 2000/1 to have been followed by a bigger study whereas we only found 8. This would reflect the opinion of most of the journal editors and experts who responded to our survey, who felt that pilot studies rarely act as a precursor for a bigger study. The main reason given was that if the pilot shows significant results then researchers may not find it necessary to conduct the main trial. In addition if the results are unfavorable or the authors find an unfeasible procedure, the main study is less likely to be considered useful. Our limited review of funding bodies was encouraging. Certainly when reviewing grant applications, we have found it helpful to have the results of a pilot study included in the bid. We think that authors of pilots studies should be explicit as to their purpose, e.g. to test a new procedure in preparation for a clinical trial. We also think that authors of proposals for pilot studies should be more explicit as to the criteria which lead to further studies being abandoned, and that this should be an important part of the proposal.

In the Portfolio Database review, only pilot studies cited an intention to estimate sample size calculations for future studies and the majority of pilot studies were full studies run with smaller sample sizes to test out a number of methodological components and clinical outcomes simultaneously. In comparison the feasibility studies tended to focus on fewer methodological components within individual studies. For example, the 6 pilot studies reported the intention to evaluate a total of 17 methodological components whereas in the 5 feasibility studies a total of only 6 methodological components were specifically identified as being under investigation (Table 4). However, both pilot and feasibility studies included trials run as complete studies, including randomization, but with sample sizes smaller than would be intended in the full study and the distinction between the two terms was not clear-cut.

Another reason for conducting a pilot study is to provide information to enable a sample size calculation in a subsequent main study. However since pilot studies tend to be small, the results should be interpreted with caution [5]. Only a small proportion of published pilot studies reported pre-study sample size calculations. Most journal editors reported that a sample size calculation is not a mandatory criterion for publishing pilot studies and suggested that it should not be done.

Some authors suggest that analysis of pilot studies should mainly be descriptive,[3,6] as hypothesis testing requires a powered sample size which is usually not available in pilot studies. In addition, inferential statistics and testing hypothesis for effectiveness require a control arm which may not be present in all pilot studies. However most of the pilot interventional studies in this review contained a control group and the authors performed and reported hypothesis testing for one or more variables. Some tested the effectiveness of an intervention and others just performed statistical testing to discover any important associations in the study variables. Observed practice is not necessarily good practice and we concur with Thabane et al [2] that any testing of an intervention needs to be reported cautiously.

The views of the journal editors, albeit from a small sample, were not particularly encouraging and reflected the experience of Lancaster et al [3]. Pilot studies, by their nature, will not produce 'significant' (i.e P < 0.05) results. We believe that publishing the results of well conducted pilot or feasibility studies is important for research, irrespective of outcome.. There is an increasing awareness that publishing only 'significant' results can lead to considerably error [7]. The journals we considered were all established, paper journals and perhaps the newer electronic journals will be more willing to consider the publication of the results from these types of studies.

We may expect that trials will increasingly be used to evaluate 'complex interventions'[8,9]. The MRC guidelines [8] explicitly suggest that preliminary studies, including pilots, be used prior to any major trial which seeks to evaluate a package of interventions (such as an educational course), rather than a single intervention (such as a drug). Thus it is likely that reviewers will be increasingly asked to pronounce on these and will require guidance as to how to review them.

## Conclusions

We conclude that pilot studies are still poorly reported, with inappropriate emphasis on hypothesis-testing. We believe authors should be aware of the different requirements of *pilot studies *and *feasibility studies *and report them appropriately. We found that in practice the definitions of feasibility and pilot studies are not distinct and vary between health research funding bodies and we suggest use of the NETSCC definition to clarify terminology.

## Competing interests

The authors declare that they have no competing interests.

## Authors' contributions

MA reviewed the papers of 2000/1 and those of 2007/8 under the supervision of MC and helped to draft the manuscript. MC conceived of the study, and participated in its design and coordination and drafted the manuscript. CC conducted the portfolio database study and commented on the manuscript. GA conducted the original study, reviewed 20 papers and commented on the manuscript. All authors read and approved the final manuscript.

## Pre-publication history

The pre-publication history for this paper can be accessed here:

http://www.biomedcentral.com/1471-2288/10/67/prepub
